# A Practical Treatise on Impotence

**Published:** 1890-12

**Authors:** 


A Practical Treatise on Impotence, Sterility and Allied Disorders of the Male Sexual Organs. By Samuel W Gross, A. M., M. D., LL. D., Professor of the Principles of Surgery and Clinical Surgery in the Jefferson Medical College of Philadelphia, etc. Fourth edition, revised by F. R. Sturgis, M. D., 8vo; pp. vii.—173. Philadelphia: Lea Brothers & Co. 1890.

This book quite maintains the reputation of the former editions of the same work. Impotence, sterility, spermatorrhea and prostatorrhea are the subjects treated. To the main text Dr. Sturgis has made valuable additions, the result of a long and varied experience in this line of surgery. The book should be read by physicians and surgeons generally.

The publishers have made the volume a most attractive one.

